# Black pleural effusion caused by pancreatic pseudocyst rupture

**DOI:** 10.1002/ccr3.1994

**Published:** 2019-01-02

**Authors:** Sho Ishigaki, Misato Kuwae, Makoto Ishii, Takanori Asakura, Soichiro Ueda, Tomoko Betsuyaku

**Affiliations:** ^1^ Division of Pulmonary Medicine, Department of Medicine Keio University School of Medicine Tokyo Japan

**Keywords:** black pleural effusion, fistula, pancreatic duct drainage, pancreatic effusion, pancreatic pseudocyst

## Abstract

The images show the path of pancreatic pleural effusion from the pancreatic pseudocyst in a patient with alcoholic pancreatitis who presented with black pleural effusion, however, without symptoms. Pancreatic pseudocyst rupture rarely causes pleural effusion; however, it should be considered in patients with chronic pancreatitis with black pleural effusion.

A 54‐year‐old man, with a 7‐year chronic alcoholic pancreatitis history, showed right pleural effusion on follow‐up computed tomography (CT). A diagnostic pleural tap revealed exudative black pleural fluid (Figure 1); the amylase level was 4752 IU/L, indicating pancreatic pleural effusion, with no evidence of bacterial infection or malignancy. On admission, a pancreatic pseudocyst, observed on CT 9 months prior, appeared shrunk (Figure 2A,B), and endoscopic retrograde cholangiopancreatography revealed pancreatic duct leakage (Figure 2C). CT revealed that the lesional fluid traversed the front of the aorta and posterior mediastinum to the right thoracic cavity via the pleura (Figure 2D,E). Hence, the patient was diagnosed with pancreaticopleural fistula and pancreatic pleural effusion and was successfully treated with thoracic and endoscopic pancreatic duct drainage.

**Figure 1:**
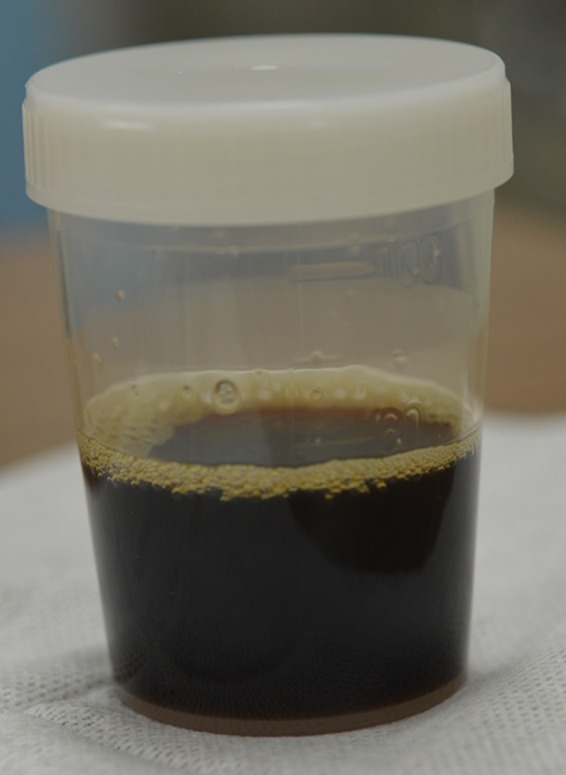
A pleural tap revealed black pleural fluid

**Figure 2:**
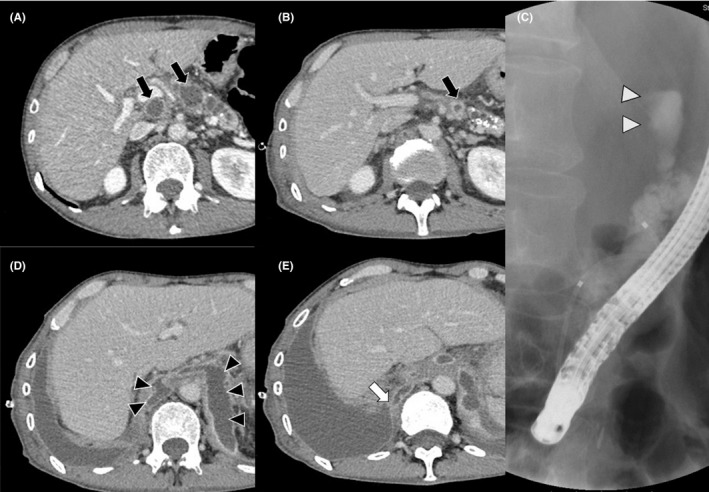
A, Nine months prior to patient admission, a pancreatic pseudocyst (black arrow) was observed on a computed tomographic scan. B, On admission, the pseudocyst had shrunk (black arrow). C, Endoscopic retrograde cholangiopancreatography showed the leakage of contrast medium from the pancreatic duct (white arrowhead). D, The fluid under the diaphragm from the pancreatic pseudocyst was observed on a computed tomographic scan. The fluid traversed the front of the aorta into the posterior mediastinum (black arrowhead). E, A computed tomographic scan showing the fluid approaching the right thoracic cavity through the pleura (white arrow)

The causes of black pleural effusion include infection, malignant melanoma, hemorrhage associated with pancreatic pseudocyst rupture, and others.^1^ The incidences of chronic pancreatitis‐associated pleural effusion with and without pancreatic pseudocyst are 4.5% and 0.4%, respectively,^2^ suggesting that pancreatic pseudocyst may be a risk factor.^1^ Here, we confirmed the pancreatic pleural effusion path from the pseudocyst using CT. Hence, pancreatic pseudocyst rupture should be considered in chronic pancreatitis associated with black pleural effusion.

## CONFLICT OF INTEREST

None declared.

## AUTHOR CONTRIBUTION

SI and MK: Primary authors, original idea, and drafting the manuscript. MI, TA, and SU: critical revision of the work. TB: supervised the project. All authors approved the final version of manuscript.

## INFORMED CONSENT

Obtained from the patient.
